# Diversity of Axonal and Dendritic Contributions to Neuronal Output

**DOI:** 10.3389/fncel.2019.00570

**Published:** 2020-01-22

**Authors:** Jean-Marc Goaillard, Estelle Moubarak, Mónica Tapia, Fabien Tell

**Affiliations:** UMR_S 1072, Aix Marseille Université, INSERM, Faculté de Médecine Secteur Nord, Marseille, France

**Keywords:** dendrite, axon, morphology, ion channels, neurotransmitter

## Abstract

Our general understanding of neuronal function is that dendrites receive information that is transmitted to the axon, where action potentials (APs) are initiated and propagated to eventually trigger neurotransmitter release at synaptic terminals. Even though this canonical division of labor is true for a number of neuronal types in the mammalian brain (including neocortical and hippocampal pyramidal neurons or cerebellar Purkinje neurons), many neuronal types do not comply with this classical polarity scheme. In fact, dendrites can be the site of AP initiation and propagation, and even neurotransmitter release. In several interneuron types, all functions are carried out by dendrites as these neurons are devoid of a canonical axon. In this article, we present a few examples of “misbehaving” neurons (with a non-canonical polarity scheme) to highlight the diversity of solutions that are used by mammalian neurons to transmit information. Moreover, we discuss how the contribution of dendrites and axons to neuronal excitability may impose constraints on the morphology of these compartments in specific functional contexts.

## Introduction

More than a century ago, *Santiago Ramon y Cajal* provided us with a tremendously extensive description of the various morphologies of the neuronal types constituting the mammalian brain and other species’ nervous systems (Cajal, 1952). From this meticulous observational work, *Cajal* hypothesized the role of the different neuronal compartments in information processing. One of his most famous contributions in that sense was the law of “dynamic polarization” that originally stated that, within a neuron, information is transmitted from the dendrites towards the soma (cellulipetal) and then in the axon away from the soma (cellulifugal). However, noticing the morphological peculiarities of several neuronal types, *Cajal* soon revisited this law, because it could only fit neuronal types where the axon directly arose from the cell body onto which all dendrites converged. In particular, *Cajal* made the observation that in many invertebrate neurons and even in some vertebrate neuronal types (such as the crook-shaped cell in the optic lobe of birds), the axon arose from a dendrite, hence compromising the theory of cellulipetal and cellulifugal propagation of information. Cajal noticed that these dendrite-emanating axons were also present in many neuronal types in mammals (e.g., the substantia nigra pars compacta dopaminergic neurons), and that other neuronal types, such as the dorsal root ganglion (DRG) neurons, presented a unipolar morphology incompatible with the first version of the dynamic polarization law. In light of these findings, *Cajal* had no choice but to reconsider the propagation of information in neurons and reformulate his law of dynamic polarization. The second version then stated that information flows towards the axis-cylinder (axipetal) away from the soma and dendrites (somafugal and dendrofugal). In doing so, *Cajal* admitted that the soma of neurons should be merely considered as *“‥.the place of the protoplasmic apparatus or the chunk of dendrite where the nucleus of the neuron sits and where chromatic inclusions are concentrated”*. Therefore, *Cajal* acknowledged that the soma is not a central compartment in terms of information transfer, but merely in terms of cellular metabolism, due to the presence of the nucleus.

In spite of *Cajal’s* caution about the division of labor in neurons, our view of neuronal function is still largely dominated by the classical “mostly passive” dendrites receiving information, and an active axon propagating and transmitting it to target neurons. However, since *Cajal’s* first observations, many more examples of “misbehaving” neurons (with a non-canonical polarity scheme) have been identified. For instance, some neurons faithfully propagate and sometimes initiate action potentials (APs) in their dendrites (oriens-alveus interneurons, midbrain dopaminergic neurons, olfactory bulb mitral cells, Gonadotropin-Releasing Hormone, GnRH neurons, DRG neurons; Hausser et al., 1995; Bischofberger and Jonas, 1997; Chen et al., 1997; Martina et al., 2000; Roberts et al., 2008; Iremonger and Herbison, 2012), some neurons release neurotransmitter from their dendrites (midbrain dopaminergic neurons, mitral cells from the olfactory bulb; Schoppa and Urban, 2003; Vandecasteele et al., 2008; Yee et al., 2019), other types of neurons have no canonical axon but produce APs and release neurotransmitter from their dendrites (retinal amacrine cells; olfactory bulb granule cells, parvalbumin and tyrosine-hydroxylase interneurons; Kosaka and Kosaka, 2008a; Wilson and Vaney, 2008; Bloomfield, 2009; Chand et al., 2015; Ona-Jodar et al., 2017; Nunes and Kuner, 2018), and some neurons possess neurites that seem to have a mixed dendrite/axon identity, such that they are called dendrons (GnRH neurons; Iremonger and Herbison, 2015). Much like *Cajal* was urged to correct the dynamic polarization law, these examples oblige us to reconsider the respective functional contributions of dendrites and axons to neuronal excitability in a case by case manner. Here, we propose to review a few cases exemplifying the diversity of dendro-axonal “solutions” used by different neuronal types. Rather than providing an exhaustive view of these variations, we will attempt to highlight the differences in functional and morphological constraints that may explain the variety of dendritic and axonal properties observed.

## Structural and Functional Definition of the Axon

Before describing the variations in the functional contribution of axons and dendrites in different neuronal types, it is important to remind what is meant when we define a given compartment as an axon or a dendrite. Dendrites and axons exhibit important differences in their anatomical, functional and structural properties.

Anatomically, axons are usually longer than dendrites and their diameter is more or less constant, even after collateral branching. In contrast, the dendritic diameter is known to taper off with distance from the soma (Craig and Banker, 1994). Moreover, the dendrites of many, but not all, mammalian neurons are covered with specialized protrusions called dendritic spines, whereas axons are considered to be devoid of spines. In addition some axons are ensheathed by myelin (produced by Schwann cells or oligodendrocytes) while dendrites are considered to be non-myelinated. Functionally, axons contain clusters of synaptic vesicles at release sites that confer them the role of the pre-synaptic compartment while dendrites, as post-synaptic compartments, generally contain essentially neurotransmitter receptors. Moreover, APs are usually initiated in the proximal part of the axon, the axon initial segment (AIS). The molecular composition of axons and dendrites also differ substantially. Dendrites essentially contain all the somatic organelles (ribosomes, endoplasmic reticulum, Golgi apparatus) while axons contain little, if any, of these components. The ion channels expressed by both compartments can also differ substantially (reviewed in Craig and Banker, 1994; Harris, 1999; Jan and Jan, 2001). One of the most important structural differences concerns the cytoskeleton composition observed in axons and dendrites: in particular, microtubules display different polarities in dendrites and axons, the latter containing only plus-end-out oriented microtubules (Baas et al., 1988; Craig and Banker, 1994). This structural peculiarity is associated with differences in microtubule dynamics, protein trafficking and Microtubule-Associated Proteins (MAP2 in dendrites, MAP1B and tau in axons) and plays a major role in neuronal polarization (reviewed in Conde and Caceres, 2009; Neukirchen and Bradke, 2011). The maintenance of neuronal polarity then depends on the establishment of the AIS in the proximal portion of the axon. The AIS constitutes an axonal subdomain that serves as: (i) a barrier that controls the mobility and diffusion of dendritic proteins along the axolemma; and (ii) a cytoplasmic selectivity filter ensuring the differential trafficking between the somatodendritic (SD) and axonal compartments (Winckler et al., 1999; Burack et al., 2000; Song et al., 2009). The AIS is characterized by an ankyrin-G and βIV-spectrin sub-membranous cytoskeletal scaffold enabling the anchoring of ion channels (sodium channels in particular) and cell-adhesion molecules (CAMs), such as Neurofascin, through its interactions with microtubules and actin filaments (Rasband, 2010). As a consequence, immunostainings of ankyrin-G, βIV-spectrin or voltage-gated sodium channels are routinely used to define the geometry of the AIS (start, end, proximal and distal parts) while the selective dendritic expression of MAP2 is often used to identify dendrites.

Therefore, important anatomical, functional and structural differences exist between dendrites and axons, which should allow us to easily distinguish these two compartments. From the perspective of the current review, we will rely mainly on structural arguments to define a given neurite as a dendrite or an axon. In this respect, it is noteworthy that most of the studies providing us with distinctive characteristics of axons and dendrites have been performed on bi- or multipolar neurons, which we will call a “classical polarity” in the next section. We will see that, depending on the neuronal type, “axonal” structural and/or functional properties can be also found in dendrites.

## Dendritic and Axonal Properties in Neurons With a “Classical Polarity”

**Figure 1:**
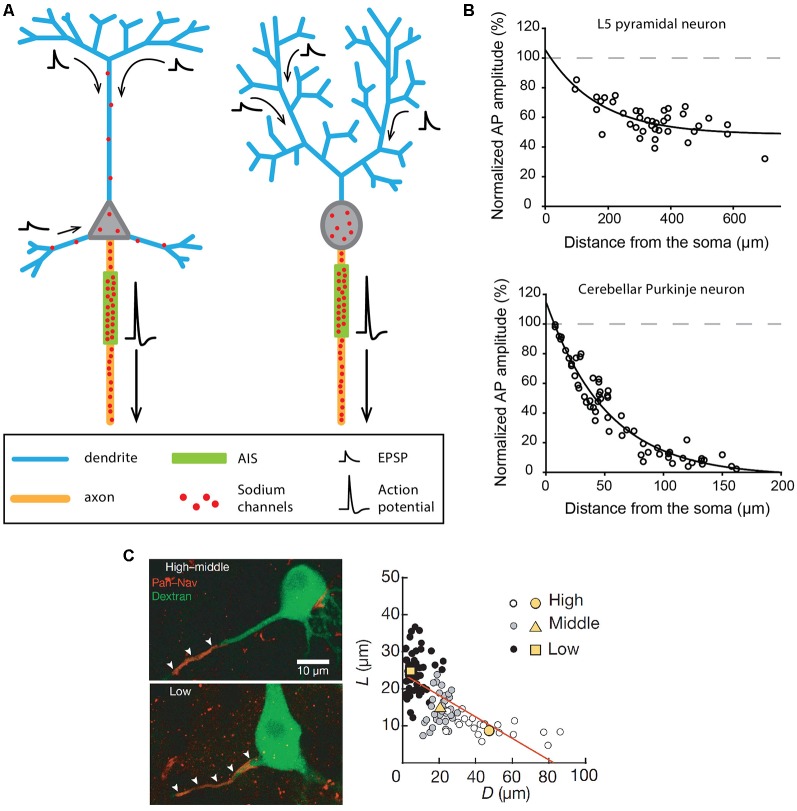
Excitability and morphological constraints of neurons with a classical polarity. **(A)** Schematics representing the morphology and excitability process in a neocortical or hippocampal pyramidal neuron (left) and a cerebellar Purkinje neuron (right). **(B)** Action potential (AP) back-propagation in L5 pyramidal neurons (top) and Purkinje neurons (bottom). **(C)** Tuning of axon initial segment (AIS) geometry as a function of preferred frequency in auditory neurons of the nucleus laminaris of birds. **(B)** Top, adapted from Stuart and Sakmann (1994); bottom, adapted from Stuart and Hausser (1994) with permission. **(C)** Reproduced from Kuba et al. (2006).

The canonical division of labor assumed for dendrites and axons is presented in Figure 1A and corresponds for instance to the behavior of cortical output neurons, such as neocortical and hippocampal pyramidal neurons or cerebellar Purkinje neurons. Although some important differences exist between these neuronal types, synaptic inputs are received by the dendrites, travel more or less passively towards the soma, and are integrated at the level of a soma-juxtaposed AIS where a high density of voltage-gated sodium channels supports the triggering of an AP (Figure 1A). In pyramidal neurons, the AP has been shown to be initiated specifically in the distal part of the AIS, at 35–45 μm from the soma (Palmer and Stuart, 2006; Kole et al., 2008; Hu et al., 2009). This privileged AP initiation site seems to be explained by: (i) its relative electrical isolation from the “current sink” effect of the soma (Brette, 2013; Thome et al., 2014; Telenczuk et al., 2017); and (ii) a high density of Nav1.6 sodium channels (Hu et al., 2009; Lorincz and Nusser, 2010) with (iii) hyperpolarized voltage sensitivities (Rush et al., 2005; Hu et al., 2009). Patch-clamp recordings, sodium imaging and immunogold labeling on neocortical and hippocampal pyramidal neurons have demonstrated that the sodium conductance density is as high as 2,500–3,000 pS/μm^2^ at the initiation site (Kole et al., 2008; Lorincz and Nusser, 2010). Both studies also concluded that sodium channel density is at least 30–40 times lower in the pyramidal cell soma and apical dendrites, consistent with the ~40 pS/μm^2^ dendritic conductance density measured by Stuart and Sakmann (1994). While in most experimental conditions this density is too low to allow the triggering of dendritic APs by incoming excitatory post-synaptic potentials (EPSPs), it underlies a partial back-propagation of AIS-initiated APs (Figure 1B; Stuart and Sakmann, 1994; Stuart et al., 1997; Vetter et al., 2001; Waters et al., 2003). In fact, a number of studies suggested that back-propagating APs might be involved in short-term and long-term synaptic plasticity mechanisms (for review, see Waters et al., 2005).

The same general behavior is observed in cerebellar Purkinje neurons, albeit with significant differences in the distribution of sodium channels (Stuart and Hausser, 1994). In fact, sodium channels are virtually absent from dendrites (≤20 pS/μm^2^), leading to very weak back-propagation of APs (Figure 1B) and to passive forward propagation of synaptic inputs (Stuart and Hausser, 1994; Roth and Häusser, 2001). In Purkinje neurons, although the AP initiation site has long been debated (Clark et al., 2005), it is now admitted that it is located in the distal AIS at a distance of 20–25 μm from the soma (Khaliq and Raman, 2006; Foust et al., 2010). Interestingly, the very short distance between the AIS and the soma in this cell type (Clark et al., 2005) is also associated with a significant density of sodium channels in the soma (Stuart and Hausser, 1994): these two conditions explain why somatic sodium channels may play an important role in modulating the frequency of AIS-initiated APs in Purkinje neurons (Khaliq et al., 2003; Khaliq and Raman, 2006).

To recapitulate, in pyramidal and Purkinje neurons, the AP is initiated in the distal AIS due to a high density of sodium channels and fails to back-propagate efficiently (although to different extents in the two cell types) due to a low density of dendritic sodium channels. Interestingly, an elegant theoretical study (Vetter et al., 2001) demonstrated that the morphological constraints imposed by the dendritic arborization in these two cell types also have a major influence on the efficiency of AP back-propagation. The densely ramified Purkinje dendrites are highly unfavorable to AP back-propagation, while the back-propagating AP linearly attenuates in the apical trunk but vanishes when entering the apical tuft of pyramidal cells (Vetter et al., 2001; Grewe et al., 2010). In some way, the density of sodium channels and the dendritic morphology have synergistic effects explaining why AP back-propagation is not faithful in these cell types.

Based on the strong differences in sodium channel density between the AIS and the dendrites in these cell types, AIS geometry (distance from the soma and length) has been postulated to have a major influence on excitability, as it may modify AP threshold or the threshold current needed to trigger an AP (Grubb and Burrone, 2010b; Bender and Trussell, 2012; Kole and Brette, 2018). Indeed, several studies have shown that chronic changes in pyramidal neuron activity (induced by KCl application, optogenetic stimulation or M-type current inhibition) were associated with a displacement of the AIS away from the soma (Grubb and Burrone, 2010a; Muir and Kittler, 2014; Wefelmeyer et al., 2015; Lezmy et al., 2017). Although the shifts in the distance were rather small (<20 μm), all studies reported parallel decreases in neuronal excitability. Along the same line, a series of beautiful studies performed on the nucleus laminaris of birds showed that the variations in AIS position and length are associated with the variation in the preferred frequency of these auditory neurons (Kuba et al., 2006, 2014; Kuba, 2012). Specifically, these authors demonstrated that high-frequency neurons displayed a significantly shorter and more distal AIS than low-frequency neurons, the middle-frequency neurons presenting an intermediate phenotype (Figure 1C; Kuba et al., 2006). The resulting differences in AP initiation site location appear to be optimized to provide the lowest AP threshold for the preferred frequency, improving the discrimination of characteristic frequencies and the detection of interaural time differences, a critical property for determining sound location. This cell-type-specific spatial tuning of the AIS is achieved in two phases during embryonic development (Kuba et al., 2014), and is partly shaped by sensory inputs, as demonstrated by the effect of sensory deprivation (Kuba et al., 2014). Interestingly, similar results have been obtained in pyramidal neurons of the mouse visual cortex, showing a developmental activity-dependent control of AIS geometry during the first post-natal weeks (Gutzmann et al., 2014).

In summary, in cell types with low SD excitability, AIS geometry (and the resulting sodium channel distribution) seems to play a predominant role in defining neuronal activity. Consistently, changes in AIS geometry in these cell types are associated with variations in neuronal excitability.

## Misbehaving Neurons

### When Dendrites Propagate and Initiate Action Potentials

**Figure 2:**
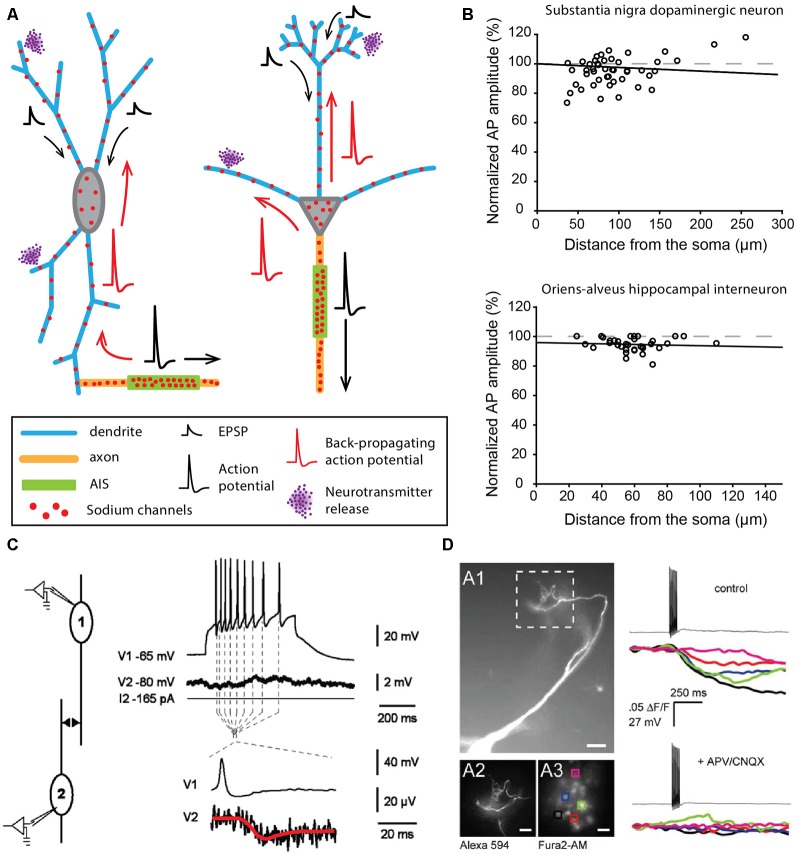
Neurons faithfully back-propagating APs and releasing neurotransmitters from their dendrites. **(A)** Schematics corresponding to a dopaminergic neuron of the substantia nigra pars compacta (left) and a mitral cell of the olfactory bulb (right). **(B)** AP back-propagation in substantia nigra dopaminergic neurons (top) and oriens-alveus interneurons (bottom). **(C)** Dendritic release of dopamine from substantia nigra dopaminergic neurons measured in paired recordings of dopaminergic neurons. The post-synaptic neuron shows a substantial hyperpolarization (red trace) in response to APs in the pre-synaptic neuron. **(D)** Dendritic release of glutamate from mitral cells of the olfactory bulb measured by calcium fluorescence in the post-synaptic cells. Mitral cells were filled with Alexa 594 (A1 panel), and regions of interest (ROI) on post-synaptic periglomerular cells (A3) were used to measure post-synaptic responses around the apical tuft of the mitral cell (12). Calcium transients were measured in all ROIs after spiking of the mitral cell (top right) and were blocked by antagonists of glutamate receptors (bottom right). **(B)** Top, adapted from Gentet and Williams (2007) © 2007 Society for Neuroscience; bottom, adapted from Martina et al. (2000), with permission. **(C)** Reproduced from Vandecasteele et al. (2008) © 2008 National Academy of Sciences. **(D)** Reproduced from Castro and Urban (2009) © 2009 Society for Neuroscience.

In contrast to the examples described above, some neuronal types display a highly excitable SD compartment, such that APs can be faithfully propagated or even initiated in dendrites in physiological conditions (Hausser et al., 1995; Bischofberger and Jonas, 1997; Chen et al., 1997; Martina et al., 2000). The contribution of the SD compartment to AP waveform was already observed in the early intracellular recordings obtained from different vertebrate neurons in the 1950s (Coombs et al., 1957a,b; Fatt, 1957). However, the first evidence for faithful dendritic back-propagation of APs was only obtained in 1995 from rat substantia nigra pars compacta dopaminergic neurons (Hausser et al., 1995). This cell type has the particularity of spontaneously generating at a regular frequency (pacemaking activity) broad biphasic APs, suggesting a strong involvement of SD sodium channels (Grace and Bunney, 1983). Two interesting observations were made in this cell type: (i) the axon mainly arises from a dendrite (axon-bearing dendrite or ABD), at distances from the soma reaching 200 μm (Figure 2A; Hausser et al., 1995; Gentet and Williams, 2007; Meza et al., 2018; Moubarak et al., 2019); and (ii) the AP recorded in different compartments of the neuron (ABD, soma or non-ABD) shows virtually no attenuation in its amplitude (Figure 2B) although it is always initiated at the AIS (Hausser et al., 1995; Gentet and Williams, 2007; Moubarak et al., 2019). This faithful back-propagation, which appears highly reliable during spontaneous pacemaking (Gentet and Williams, 2007; Blythe et al., 2009; Moubarak et al., 2019) may be abolished under specific conditions, for instance when barrages of EPSPs onto the ABD are used to trigger firing or when dopamine is used to dampen somatic excitability (Gentet and Williams, 2007). Since the seminal observation of Hausser et al. (1995), faithful back-propagation has been described in at least two other mammalian cell types, the olfactory bulb mitral cells (Figure 2A; Bischofberger and Jonas, 1997; Chen et al., 1997; Xiong and Chen, 2002) and the oriens-alveus interneurons of the hippocampus (Figure 2B; Martina et al., 2000). Interestingly, these three cell types share a common property, in that they express a rather high density of sodium channels at the dendritic level: the measured conductance density is ~75, ~80 and ~110 pS/μm^2^ in dopaminergic neurons (Moubarak et al., 2019), mitral cells (Bischofberger and Jonas, 1997) and oriens-alveus interneurons (Martina et al., 2000), respectively. Moreover, these three cell types present morphological peculiarities favoring AP back-propagation. In mitral cells, the primary dendrite is mainly unbranched and of constant diameter (Shepherd, 1966), thus limiting low-safety points for current spread. The secondary dendrites have also few branching points and are therefore favorable to AP propagation (Price and Powell, 1970a; Xiong and Chen, 2002). Along the same line, Vetter et al. (2001) demonstrated that the rather simple dendritic arborization of dopaminergic neurons (with much less branching points than pyramidal or Purkinje neuron dendrites) favors faithful back-propagation of APs, even for low densities of dendritic sodium conductances. Interestingly, oriens-alveus interneurons exhibit a dendritic morphology very similar to dopaminergic neurons, with a large soma, short and seldom branched dendrites and a dendrite-emanating axon (McBain et al., 1994; Hausser et al., 1995; Hajos and Mody, 1997; Martina et al., 2000; Moubarak et al., 2019). Therefore in these three cell types, dendritic morphology and SD sodium channel density may have synergistic effects favoring faithful back-propagation of the AP in the entire dendritic arborization.

In addition to faithfully back-propagating APs, mitral cells and oriens-alveus interneurons are also able to initiate full APs from the dendrites in specific experimental conditions (Chen et al., 1997, 2002; Martina et al., 2000). In mitral cells, AP initiation occurs in the distal apical dendrite when: (i) the soma is transiently inhibited by local interneurons (Chen et al., 1997) or (ii) when a strong glutamatergic input onto the distal dendrite is recruited (Chen et al., 2002). This second mode of triggering of dendritic initiation is reminiscent of the oriens-alveus interneurons, where brief high-intensity stimulation has to be used to displace the initiation site from the AIS to the non-axon-bearing dendrite (Martina et al., 2000). What may be the role of dendritic initiation in these two cell types? In mitral cells, synaptic inputs are strongly compartmentalized, with excitatory olfactory nerve inputs impinging specifically on the distal tuft of the primary dendrite and local inhibitory inputs projecting onto secondary dendrites near the soma (for review, see Schoppa and Urban, 2003). While AIS geometry has not been closely examined in this cell type, the axon seems to always arise from the soma, with a rather proximal AIS (Lorincz and Nusser, 2008). The existence of a dendritic initiation site remote from the soma may ensure that responses to sensory inputs persist in the presence of strong inhibition of the soma and AIS by local interneurons (Chen et al., 1997). Such a clear segregation of inputs does not seem to be present in oriens-alveus interneurons, where the axon can arise either from a subiculum- or a CA3-oriented dendrite (Martina et al., 2000). The sensitivity of dendritic initiation to strong excitatory inputs suggests that it may ensure fast and reliable activation of these neurons. Alternatively, it could be important for the induction of long-term changes in synaptic efficacy (Martina et al., 2000). In contrast to these two cell types, dendritic initiation of APs has so far not been observed in dopaminergic neurons. As suggested by several publications, the presence of a high density of sodium channels in the SD compartment may not only serve AP back-propagation in this cell type but also play a central role in the generation of regular spontaneous firing (Wilson and Callaway, 2000; Tucker et al., 2012; Jang et al., 2014; Moubarak et al., 2019). Pharmacological blockade, dynamic-clamp experiments and computational modeling indeed suggest that SD sodium channels control pacemaking frequency (Tucker et al., 2012; Jang et al., 2014; Moubarak et al., 2019), even though the AP is always initiated at the AIS. Interestingly, oriens-alveus interneurons have also been shown to generate a spontaneous pacemaking pattern of activity *in vitro* (McBain et al., 1994), involving, in particular, the H-type current (carried by HCN channels) as a source of depolarization (Maccaferri and McBain, 1996). In substantia nigra dopaminergic neurons, pacemaking in juvenile neurons has been postulated to be HCN and sodium channel-dependent (Chan et al., 2007). The expression of a high density of both types of channels in the dendrites of oriens-alveus interneurons (Maccaferri and McBain, 1996; Martina et al., 2000) suggests that pacemaking activity might be generated in a similar way in this neuronal type. Knowing that these interneurons project a densely branched axon onto the apical dendrites of CA1 pyramidal cells (McBain et al., 1994; Martina et al., 2000), these data suggest that oriens-alveus interneurons may be able to provide a tonic inhibition of CA1 pyramidal dendrites in the absence of synaptic drive (Maccaferri and McBain, 1996).

In summary, we provided examples demonstrating that, in cell types with a high density of SD sodium channels, APs can be faithfully propagated in the entire dendritic arborization and sometimes be initiated at the dendritic level. While the functional constraints explaining the need for a highly excitable SD compartment may vary between cell types, the next section suggests that it may be necessary when dendrites fulfill another “axonal” function: releasing neurotransmitters.

### When Dendrites Release Neurotransmitters

The SD release of dopamine (DA) by midbrain dopaminergic neurons was first demonstrated in the late 70s in both acute midbrain slices and *in vivo* (Geffen et al., 1976; Nieoullon et al., 1977). In parallel, dendro-dendritic synapses containing DA-filled vesicles were observed between neighboring DA neurons (Wilson et al., 1977). More recently electrophysiological measurements demonstrated that the SD release of DA was associated with hyperpolarization of the post-synaptic neuron (Figure 2C; Beckstead et al., 2004; Vandecasteele et al., 2008). Although many proofs of the SD release of DA between neighboring dopaminergic neurons have been gathered, the details about the release mechanisms are still debated (for review, see Ludwig et al., 2017; Gantz et al., 2018). What is clearly admitted is that DA released at dendro-dendritic synapses binds to D2 receptors on the post-synaptic neuron, which triggers a hyperpolarization mainly due to GIRK channel activation (Beckstead et al., 2004). This release appears to depend at least partly on back-propagating APs (Vandecasteele et al., 2008) and on extracellular Ca^2+^ (Yee et al., 2019) and would involve vesicular and/or tubulo-vesicular release of DA (Groves and Linder, 1983; Nirenberg et al., 1996a,b; Bergquist et al., 2002; for review, see Ludwig et al., 2017). Interestingly, a recent study suggested that bursts of APs may fail to faithfully back-propagate to the entire dendritic arborization (Gentet and Williams, 2007), suggesting that dendro-dendritic release of DA would not follow high-frequency discharge of APs, in contrast with the documented potentiation of axonal DA release in the striatum during bursting patterns of activity of midbrain dopaminergic neurons (Gonon, 1988; Heien and Wightman, 2006; Zweifel et al., 2009). This suggests that, at least in some conditions, axonal and SD release of DA might be dissociated. It is noteworthy that the axonal and dendritic release sites are separated by several millimeters in the rodent brain. In addition, the dendro-dendritic release of DA onto neighboring GABAergic neurons has also been functionally described and would involve D1 receptors coupled to TRPC3 ion channels, resulting in an increase of activity of the post-synaptic target (Zhou et al., 2009).

The olfactory bulb is a region where multiple cell types seem to use the dendritic release of neurotransmitters (for review, see Schoppa and Urban, 2003, see also next *“When Dendrites do all the Job: Neurons Lacking a Canonical Axon”* section). Among these, the dendro-dendritic synapses formed by mitral cells onto other types of neurons are the best documented (Schoppa and Urban, 2003). The synapses between mitral cells and granule cells were the first dendro-dendritic synapses described (Hirata, 1964), and their functional role in the processing of olfactory information was identified early on by the electrophysiological and computational studies of Rall et al. (1966). These synapses are formed by the secondary dendrites of the glutamatergic mitral cells contacting specific dendritic structures (gemmules) of the GABAergic granule cells. Interestingly, these dendro-dendritic synapses are reciprocal in 90% of the cases, providing very fast feedback inhibition in response to mitral cell activation (Shepherd, 1966; for review, see Schoppa and Urban, 2003; Shepherd et al., 2007). Moreover, as APs are back-propagating faithfully along mitral cell secondary dendrites (Xiong and Chen, 2002) and each granule cell contacts several mitral cells (for review, see Shepherd et al., 2007), these synapses allow long-range lateral inhibition *via* the activation of granule cells. Interestingly, mitral cells also make dendro-dendritic synapses with another type of interneurons, the periglomerular cells, *via* their primary dendrite (Figure 2D; for review, see Schoppa and Urban, 2003; Nagayama et al., 2014). These dendro-dendritic synapses made by mitral cells play an essential role in olfactory processing as they mediate interglomerular (mitral-granule) and intraglomerular inhibition (mitral-periglomerular), respectively (Schoppa and Urban, 2003; Nagayama et al., 2014). The synchronous activation of mitral cells belonging to the same glomerulus is also important for olfactory processing, and evidence has been found that overlapping mitral cells can be coupled by reciprocal excitation (Schoppa and Westbrook, 2002; Urban and Sakmann, 2002). Interestingly, this excitation would be mediated by glutamate released at the apical tuft of the primary dendrite and activating neighboring synapses by spillover (Schoppa and Westbrook, 2002). It must be noted that tufted cells, another group of excitatory projection neurons located in a different layer than mitral cells, present very similar patterns of dendro-dendritic interactions with olfactory interneurons (Schoppa and Urban, 2003; Nagayama et al., 2014).

Dopamine, glutamate, and GABA are not the only neurotransmitters to be released from dendrites. Dendritic release of the neuropeptides oxytocin and vasopressin by the magnocellular neurons of the supraoptic and paraventricular nuclei has been shown to play a critical role in the regulation of activity of this neuronal population (Ludwig et al., 2002; for review, see Ludwig and Leng, 2006; Ludwig and Stern, 2015; Ludwig et al., 2017). Somata and dendrites of these neurons have been shown to contain large amounts of neurosecretory granules (Pow and Morris, 1989) and SD release is induced by Ca^2+^ increase (Ludwig et al., 2002). SD release is not strictly dependent on APs, suggesting that axonal release in the neurohypophysis and SD release might be sensitive to different stimuli and regulated separately, at least to some extent (Ludwig, 1998; Wotjak et al., 1998; Ludwig et al., 2002). Moreover, this release does not seem to occur at well-defined synapses (Pow and Morris, 1989). The SD released neuropeptides would exert their effects mainly by autocrine and paracrine actions, which are allowed by the long-lasting half-lives of these peptides in the cerebrospinal fluid (Mens et al., 1983). Oxytocin and vasopressin released from the dendrites of magnocellular neurons are thought to exert powerful self-regulatory actions, inhibiting or promoting the activity of the neurons releasing them on a long-term range (Wotjak et al., 1998; for review, see Ludwig and Leng, 2006; Ludwig and Stern, 2015; Ludwig et al., 2017).

In summary, we provided examples showing that several types of neurons are releasing neurotransmitters from their dendritic compartment. In the cases discussed here, it seems that long-range projecting neurons rely on dendritic release to exert a local control on activity: dendritic dopamine release inhibits neighboring dopamine neurons (and may also have an inhibitory autocrine effect on the releasing neuron), dendritic glutamate release by mitral cells produces short-range lateral inhibition *via* the activation of interneurons and dendritically released oxytocin and vasopressin exert a self-regulatory action on magnocellular neuron activity. Interestingly, the differences in release mechanisms between the axon and the dendrites (magnocellular neurons) or the possibility to gate back-propagating APs (dopamine neurons, mitral cells) seem to provide these cell types with the possibility to control independently axonal and dendritic release of neurotransmitter, hence considerably expanding the computational repertoire of these cell types. In contrast, we will see now that the functional impact of some interneurons can be restricted to very local actions by the total absence of an axon.

### When Dendrites do all the Job: Neurons Lacking a Canonical Axon

The first observation of axonless cells was made *by Camillo Golgi* in the mid-1870s on the mammalian olfactory bulb: *Golgi* identified small cells in the mitral cell body layer exhibiting long branching dendrites but apparently lacking an axon (for review, see Shepherd et al., 2007). *Golgi* was one of the main supporters of the reticular theory stating that nerve cells are all connected *via* a continuous network of axon collaterals. As a consequence, *Golgi* questioned the “nervous” identity of these newly identified cells. The neuronal identity of axonless granule cells of the olfactory bulb was later inferred by *Cajal* who suggested that granule cells could spread excitatory signals between mitral cells through “avalanche conduction” (for review, see Shepherd et al., 2007). Despite the early observation that neurons without an axon do exist in the mammalian brain, little work was done on these peculiar cells and their physiology until the late 1960s. Since then, several other axonless neurons have been identified in vertebrate (Price and Powell, 1970b,c; Toida et al., 1994; Shepherd et al., 2007; Wilson and Vaney, 2008; Bloomfield, 2009) and invertebrate nervous systems (Laurent et al., 1993; Laurent, 1993; Wilson and Laurent, 2005), and seem to be mainly present in sensory systems such as the visual or the olfactory systems. Although axonless neurons in invertebrates appear to be mainly non-spiking neurons (Laurent et al., 1993; Laurent, 1993; Wilson and Laurent, 2005), it is not the case for vertebrate axonless neurons, which seem to fire APs and participate in network activity *via* the dendro-dendritic release of neurotransmitters.

**Figure 3:**
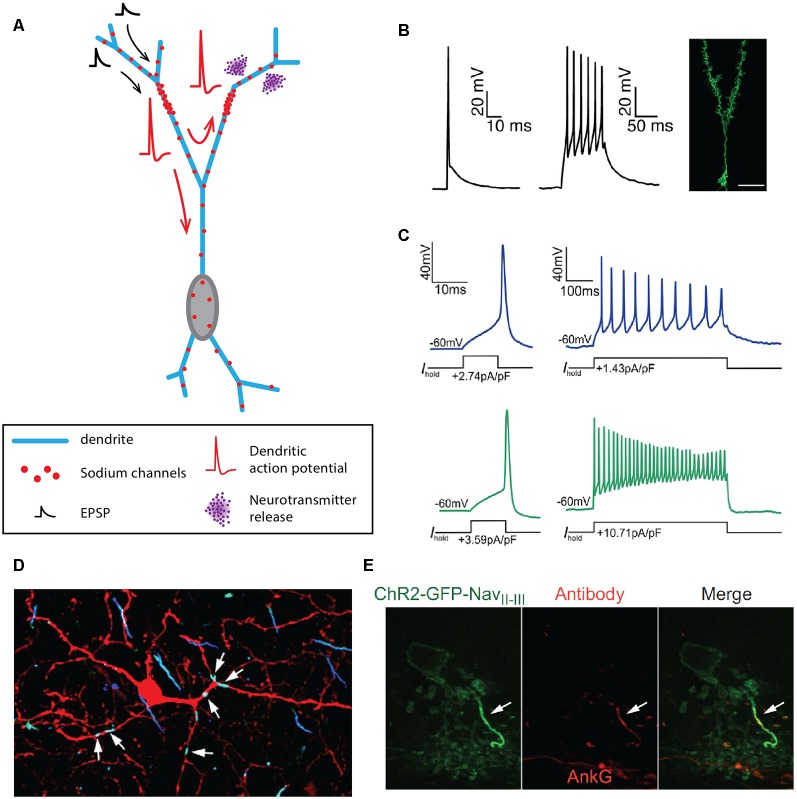
Axon-less neurons. **(A)** Schematic representing the morphology of an axonless granule cell of the olfactory bulb. **(B)** Dendritic APs emitted by granule cells of the olfactory bulb. **(C)** APs recorded at the soma of axonless (blue traces) and axonic (green traces) juxtaglomerular tyrosine hydroxylase interneurons. **(D)** Six hotspots of sodium and ß-spectrin are identified on the dendrites of a rat parvalbumine interneuron of the olfactory bulb. **(E)** A single cluster of Nav1.1 sodium channels and ankyrin-G (AnkG) is found in AII amacrine cells of the retina. **(B)** Reproduced from Nunes and Kuner (2018). **(C)** Reproduced from Chand et al. (2015). **(D)** Reproduced from Kosaka et al. (2008); with permission. **(E)** Reproduced from Wu et al. (2011).

In the olfactory bulb, three axonless neuronal types have been identified: the granule cells (Figure 3A), the parvalbumine interneurons of the external plexiform layer (EPL) and the juxtaglomerular tyrosine hydroxylase interneurons (Rall et al., 1966; Toida et al., 1994; Chand et al., 2015). Unlike what Cajal initially suggested, granule cells of the olfactory bulb are in fact inhibitory interneurons, releasing GABA on mitral cell secondary dendrites through reciprocal dendro-dendritic synapses (Rall et al., 1966; Rall and Shepherd, 1968; Price and Powell, 1970b,c). Granule cell dendrites express Nav1.2 clusters throughout the cell surface, allowing the generation and propagation of full dendritic APs in response to current injection or stimulation by odorants (Figure 3B, Egger et al., 2003; Margrie and Schaefer, 2003; Zelles et al., 2006; Egger, 2008; Nunes and Kuner, 2015, 2018; Ona-Jodar et al., 2017). Nav1.2 channel deletion leads to the abolition of AP generation and dendritic GABA release, consequently removing mitral cell inhibition and delaying odor discrimination (Nunes and Kuner, 2018). Parvalbumine interneurons also seem to release GABA onto mitral and tufted cells in the EPL *via* reciprocal synapses (Toida et al., 1994, 1996), and electrophysiological recordings of unidentified “axonless” interneurons in the EPL suggested that these cells were able to fire APs under current injection (Hamilton et al., 2005). Interestingly, in the case of the juxtaglomerular tyrosine hydroxylase interneurons, only a fraction of the cells seems to be deprived of an axon, even though stainings against specific axonal markers were not performed to ascertain the total absence of an axon (Kosaka and Kosaka, 2008b; Chand et al., 2015; for review, see Kosaka and Kosaka, 2016). The axonic and axonless subpopulations can be distinguished: (i) morphologically, as the axonless neurons exhibit a smaller soma and a shorter dendritic arborization than their axonic counterpart but also (ii) functionally, as axonic interneurons appear to be more excitable and generate biphasic APs while axonless interneurons are less excitable and fire monophasic APs (Figure 3C, Chand et al., 2015; Galliano et al., 2018). Both axonic and axonless tyrosine hydroxylase interneurons are able to release GABA and DA from their dendrites (Borisovska et al., 2013; Vaaga et al., 2017; for review, see Kosaka and Kosaka, 2016).

In the retina, the main type of axonless neurons seems to be the AII amacrine cells, a class of interneurons mediating day and night vision by transferring information from the rod bipolar cells to the ON and OFF cone pathways *via* electrical synapses and dendritic release of glycine, respectively (Strettoi et al., 1992; Tsukamoto et al., 2001; for review, see Bloomfield and Dacheux, 2001). These unipolar neurons exhibit two levels of dendritic branching reaching proximally the ON-sublamina and more distally the OFF-sublamina. Rod information is transmitted to AII amacrine cells *via* electrical synapses (Tsukamoto et al., 2001), AII amacrine cells then amplify and accelerate the post-synaptic response through SD Nav1.1 channels (Tian et al., 2010; Wu et al., 2011) and the initiation of small and broad sodium spikelets (<10 mV and >5 ms) in the primary dendritic level (Boos et al., 1993; Tamalu and Watanabe, 2007; Cembrowski et al., 2012). These sodium spikelets could act as a threshold mechanism to selectively amplify rod signals (Smith and Vardi, 1995; Tian et al., 2010) and potentially affect AII amacrine cells’ output to the ON and OFF cone pathways, although it is important to note that this output persists under TTX application (Tian et al., 2010). This last finding suggests that, in specific cases, compact axonless vertebrate neurons may be able to transmit information in the absence of APs, similar to what is known for invertebrate axonless neurons (Laurent et al., 1993; Laurent, 1993; Wilson and Laurent, 2005).

One question that arises from the observation of axonless neurons is whether the AP is still generated from a preferred site in the dendritic tree? Although the mechanisms of AP initiation in most of these cell types are poorly understood, some evidence suggests the presence of one or more AIS-like compartments in the dendrites of parvalbumine interneurons and potentially granule cells of the olfactory bulb (Kosaka and Kosaka, 2008a; Kosaka et al., 2008), and AII amacrine cells of the retina (Wu et al., 2011). Indeed, immunohistochemical studies by Kosaka et al. (2008) revealed that each parvalbumine interneuron could exhibit 2–7 clusters of βIV-spectrin, sodium channels and ankyrin-G along their dendrites (Figure 3D). These clusters measured between 3 and 28 μm and could be located up to 50 μm away from the soma, on a 1st to 9th order dendrite (Kosaka and Kosaka, 2008a; Kosaka et al., 2008). Although they did not study olfactory bulb granule cells directly, the authors also mentioned that multiple AIS-like hotspots could also be observed on their dendrites (Kosaka et al., 2008). The idea of multiple sites for AP initiation in granule cells is also supported by the variability in amplitude of somatically recorded APs, which could be a consequence of the morphological characteristics of the specific branch it was generated from, as suggested by Zelles et al. (2006), Egger (2008) and Nunes and Kuner (2018). Unlike olfactory bulb axonless neurons, AII amacrine cells exhibit a single hotspot containing Nav1.1, Neurofascin and ankyrin-G (Figure 3E, Wu et al., 2011). Moreover, disrupting the AIS-targeting motif of Nav1.1 channels results in the abolition of firing, confirming the implication of this AIS-like compartment in AP generation (Wu et al., 2011).

Interestingly, in several brain regions (olfactory bulb, neocortex, striatum), neurons produced during adulthood (adult neurogenesis) are devoid of an axon (Kosaka and Kosaka, 2009; Le Magueresse et al., 2011; Inta et al., 2015; Galliano et al., 2018). For instance, calretinin-positive interneurons with a granule cell-like morphology and a single primary dendrite are produced in the striatum by post-natal neurogenesis (Inta et al., 2015). The post-natally born neocortical axonless neurons (GABA CR+/5HT3A interneurons) also display a granule cell-like morphology and make dendro-dendritic synapses (Le Magueresse et al., 2011). In the olfactory bulb, while both axonic and axonless tyrosine hydroxylase interneurons are produced by embryonic and perinatal neurogenesis, adult neurogenesis only produces axonless neurons (Kosaka and Kosaka, 2009; Galliano et al., 2018).

In summary, some neurons involved in local treatment of information in sensory systems seem to have evolved to carry out their function without the need of a specific output compartment, the axon. While in invertebrates the axonless neurons do not rely on APs and use graded synaptic transmission, many of the vertebrate axonless neurons display dendritic features usually considered to be “axon-specific” such as ankyrin-G expression and high-density clusters of sodium channels allowing the dendritic initiation of APs and subsequent dendritic transmitter release.

### When Dendrite and Axon Are the Same Neurite

**Figure 4:**
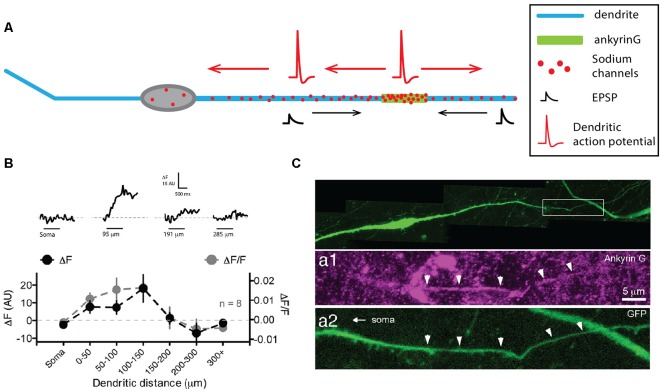
Gonadotropin-releasing hormone (GnRH) neurons display a dendron with mixed axonal and dendritic properties. **(A)** Schematic representing the simplified morphology of a GnRH neuron. The dendron appears on the right as a thicker dendrite. **(B)** Sodium fluorescence imaging shows that maximum sodium influx occurs at 100–150 μm from the soma. Top, sodium fluorescence signal (ΔF) recorded at four different locations in the same neuron in response to 1 s depolarization-induced firing of the soma (horizontal bars). Bottom, average ΔF (black) and ΔF/F for eight neurons recorded at seven different locations show that sodium transients are maximal in the first 150 μm. **(C)** The dendron displays ankyrin-G staining at ~90 μm from the soma. Top, green fluorescent protein (GFP) staining of a GFP-expressing GnRH neuron. Expanded view of the dendron of the neuron shown in the top image, illustrating the presence of ankyrin-G staining (purple, middle image) colocalized with the GFP staining (green, bottom image). **(B)** Modified from Iremonger and Herbison (2012). **(C)** Adapted from Herde et al. (2013).

The hypothalamic GnRH neurons do not really fall into any of the categories that we described so far, as they seem to not have well-distinguished dendrites and axon, but instead, display a neurite with mixed properties hence named the “dendron” (Figure 4A, for review, see Iremonger and Herbison, 2015). GnRH neurons lie in the medial septum, rostral pre-optic area and anterior hypothalamic area and project to the median eminence where the release of GnRH regulates luteinizing and follicle-stimulating hormone release from the anterior pituitary. These neurons most often have a very simple fusiform/bipolar morphology with unbranched neurites arising from opposite sides of the soma (for review, see Silverman et al., 1994). Interestingly, the lack of immunostaining against classical axonal proteins (Herde et al., 2013) and the presence of spines (Campbell et al., 2005) indicate that both of these processes are dendrites, even though they are not labeled by anti-MAP2 stainings (Herde et al., 2013). In most GnRH neurons, at least one of these primary dendrites projects to the median eminence over considerable lengths (>1,000 μm, Campbell et al., 2005, 2009; Herde et al., 2013; Herde and Herbison, 2015), displaying spines over its whole length, although spine density within the first 50 μm from the soma is much higher than at more remote locations (Campbell et al., 2005). Since no axon could be found in these neurons, several groups wondered whether these dendrites were capable of initiating and propagating APs (Roberts et al., 2008; Campbell and Suter, 2010; Iremonger and Herbison, 2012; Herde et al., 2013). Indeed, using double soma-dendrite recordings, Roberts et al. (2008) demonstrated that APs could back-propagate from the soma to the dendrites and that spontaneous dendritic AP initiation occurred in GnRH neurons. The use of Na^+^-sensitive dyes and dendritic recordings confirmed the faithful propagation of APs and suggested that the spike initiation site is located within the first 200 μm of one of the two dendrites (Figure 4B, Iremonger and Herbison, 2012). Consistently, ankyrin-G stainings labeled a short segment located on average 90 μm away from the soma on the dendrite projecting towards the median eminence (Figure 4C, Herde et al., 2013), leading these authors to name this process “dendron” (Herde et al., 2013; Herde and Herbison, 2015; Iremonger and Herbison, 2015). Interestingly, a more recent study suggested that 40% of GnRH neurons indeed possess an axon, although they also display a dendron projecting towards the medial eminence (Herde and Herbison, 2015). Surprisingly, despite the presence of an axon, ankyrin-G staining was overall predominantly located on a dendrite (75% of the time). Since the targets of the GnRH axon are currently unknown, while dendrons projecting onto the median eminence have been clearly identified (Herde et al., 2013), it is still currently assumed that the dendron is the main output of GnRH neurons involved in the control of luteinizing and follicle-stimulating hormone release in the anterior pituitary.

In summary, although recent evidence suggested that GnRH neurons can possess an axon, the functional output of these neurons seems to substantially rely on a long-range projecting spiny dendrite that can initiate and faithfully propagate APs, due to the presence of ankyrin-G and clustering of sodium channels, and has therefore been considered to be neither an axon nor a dendrite, but a dendron.

### The Specific Case of Unipolar Neurons

**Figure 5:**
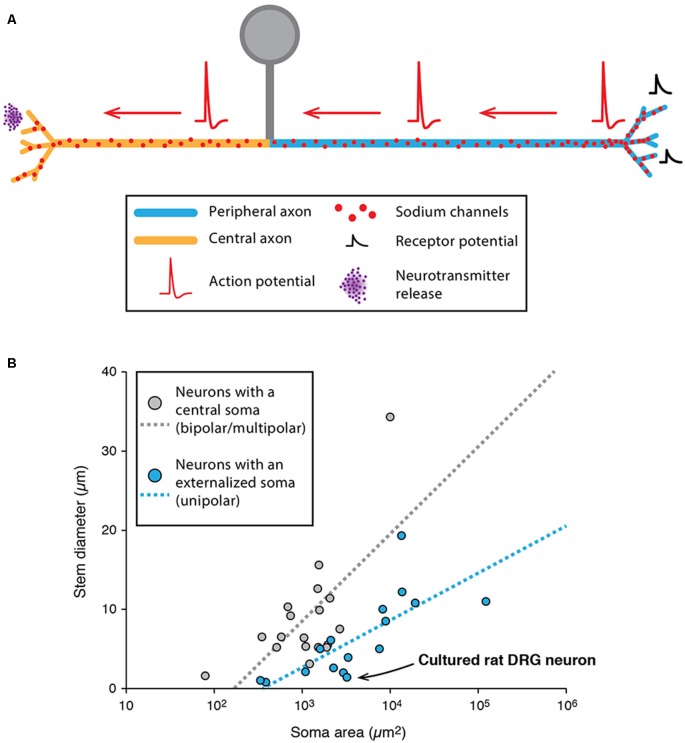
Specificities of unipolar neurons. **(A)** Schematic representing the simplified morphology of a dorsal root ganglion (DRG) neuron. **(B)** Scatter plot showing the relationship between soma area and stem neurite diameter in neurons with a central soma (gray dots) and neurons with an externalized soma (unipolar neurons, blue dots). The regression lines showing the correlation between the soma area and stem neurite diameter appear as dotted lines of matching colors. The point corresponding to cultured rat DRG neurons is indicated with a black arrow. **(B)** Adapted from Hesse and Schreiber (2015); with permission.

Unipolar neurons are defined as having a single neurite arising from the soma. At least one type of neurons in mammals is unipolar: the DRG neurons (Cajal, 1952). The DRG neurons constitute the first step of sensory pathways, conveying information about pain, temperature, proprioception, and mechanoreception. From an anatomical point of view, these neurons are unipolar, with a stem axon leaving the soma and two axonal branches projecting to the periphery and to the spinal cord, respectively (Figure 5A, for review, see Nascimento et al., 2018). Both branches are myelinated (for myelinated DRG neurons), the AP being initiated in the peripheral branch and conducted towards the spinal cord. However, several elements suggest that the peripheral branch might be more of a dendrite and that the “pseudo-unipolarity” of DRG neurons is in fact acquired during development (Nascimento et al., 2018). *Cajal* was the first one to describe in detail this process when he observed that bird DRG neurons are indeed bipolar during embryonic development and only become secondarily unipolar (Cajal, 1952), acquiring this peculiar morphology where the peripheral dendritic and central axonal branches are directly connected to each other. While the subject of the subcellular nature of the stem axon is still debated, one hypothesis is that it corresponds to a shrinking and elongation of the somatic membrane that would bring the dendritic and axonal branches in close apposition (Nascimento et al., 2018). Concerning the AIS, while several studies have observed a distinctive AIS in cultured embryonic DRG neurons (Zhang and Bennett, 1998; Dzhashiashvili et al., 2007; Hedstrom et al., 2007), there is no evidence of a well-defined AIS in adult DRG neurons *in vivo* (Nascimento et al., 2018). From a functional point of view, APs are initiated in the peripheral branch close to the peripheral endings where sensory transduction occurs (Carr et al., 2009), although the AP initiation site can slightly move depending on the level of hyperpolarization of the terminal. Consistent with this finding, in non-myelinated peripheral branches, Nav1.8 and Nav1.9 sodium channels were found to be distributed homogeneously in the peripheral endings (Black and Waxman, 2002).

While unipolar neurons represent an exception in the mammalian nervous system, it is interesting to note that most arthropod neurons are unipolar (Sánchez-Soriano et al., 2005; Rolls et al., 2007; Rivera-Alba et al., 2014; Triarhou, 2014; Hesse and Schreiber, 2015; Niven, 2015). In insects and crustaceans, most neurons have an “externalized” cell body from which emerges a single “stem” neurite giving rise to both dendrites and axons (Hesse and Schreiber, 2015; Niven, 2015). This organization seems to bear several advantages because: (i) it allows a clear segregation of a cell body area and a pure neuropil area where contacts are made between pre-synaptic and post-synaptic neurons (Rivera-Alba et al., 2014); and (ii) it removes an electrotonically unfavorable compartment (the soma) from the signal propagation path (dendrite to axon), thus preventing unnecessary signal attenuation (Hesse and Schreiber, 2015). In fact, two studies suggested that soma size may be one of the main constraints determining whether the soma is externalized (leading to a unipolar morphology), independent of the species studied (Rivera-Alba et al., 2014; Hesse and Schreiber, 2015). More precisely, using computational modeling of passive and active propagation of signals, Hesse and Schreiber (2015) demonstrated that soma externalization is beneficial only if the stem neurite is sufficiently resistive (in terms of axial resistance) compared to soma size. Indeed, data collected from various species (including insects, crustaceans and mammals) seem to confirm this conclusion, as the ratio stem neurite/soma size is significantly smaller in neurons displaying externalized somata (Figure 5B). Interestingly, the soma of DRG neurons is very large (20–100 μm in rats) and the ratio stem axon/soma size follows the rule suggested by Hesse and Schreiber (2015; see Figure 5B). So far, it is still unclear why unipolar neurons are predominant in arthropods while multipolar neurons represent the vast majority of vertebrates and lower invertebrates (Bullock and Horridge, 1965; Triarhou, 2014). Interestingly, insect unipolar neurons have been shown to “regress” to a bipolar or multipolar morphology after a few days in culture (Sánchez-Soriano et al., 2005), similar to what has been observed for DRG neurons. One hypothesis for cell body externalization in higher invertebrates is that the cell body is displaced out of the neuropil area, and neurons become unipolar only during this process (Sánchez-Soriano et al., 2005). Therefore, while invertebrate neurons appear rather dissimilar to vertebrate neurons, this dissimilarity appears to be secondarily acquired during development due to cell body exclusion from the connecting path. Moreover, recent studies suggest that their dendrites and axons behave very much like their vertebrate counterparts (Sánchez-Soriano et al., 2005; Rolls et al., 2007). Consistent with this, although the presence of a distinctive AIS in invertebrates has long been questioned, recent evidence demonstrated that cultured drosophila neurons express a specific isoform of ankyrin in the proximal axonal region (Rolls et al., 2007; Jegla et al., 2016), much like ankyrin-G in vertebrate neurons.

In summary, many neurons in the animal kingdom are unipolar, departing from the classical neuronal morphology depicted in most textbooks. In spite of this particularity, recent evidence suggests that invertebrate unipolar neurons possess dendrites and axons with clearly segregated functions and specific molecular signatures (Sánchez-Soriano et al., 2005; Rolls et al., 2007; Rolls and Jegla, 2015; Jegla et al., 2016). On the other side, vertebrate DRG neurons display two neurites behaving like axons, even though their developmental origin suggests that the peripheral and central branches are a dendrite and an axon, respectively (Cajal, 1952; Nascimento et al., 2018).

## Are Dendritic and Axonal Properties Co-tuned?

So far, we reviewed examples demonstrating the diversity of morphological and functional properties of dendrites and axons in various neuronal types. Some neurons display a highly excitable axon together with fairly passive dendrites. In other neuronal types, dendrites are highly excitable and can initiate, propagate APs and release neurotransmitters. In the olfactory bulb and the retina, some interneurons are devoid of an axon, and all pre- and post-synaptic functions are carried out by the dendrites. Finally, some neurons, such as the DRG neurons, possess neurites that all behave more or less like axons.

From these observations, one may wonder whether general rules bind variations in dendritic excitability and morphology to variations in axon or AIS excitability and morphology. In other words, are dendritic and axonal properties balancing each other to ensure optimal neuronal output, such that axonal properties might differ between neurons with passive or active dendrites? We will see that in some neuronal types, dendritic and axonal properties seem to be co-tuned to optimize neuronal output, while in others axonal and dendritic properties appear fairly independent from each other. Since many morphological and biophysical parameters can influence both dendritic and axonal properties, these relationships are particularly difficult to demonstrate experimentally. Therefore, computational approaches have proved particularly useful in determining why co-tuning rules might exist or be absent from a specific neuronal type.

### Evidence of Co-tuning of Axonal and Dendritic Properties

**Figure 6:**
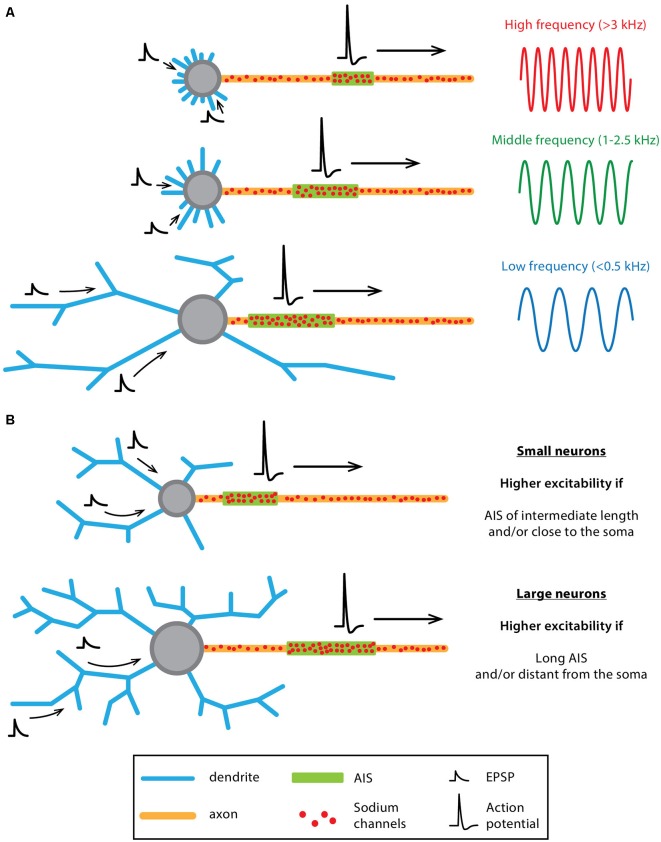
Co-tuning of somatodendritic (SD) and AIS morphologies influences neuronal output in “classical polarity” neurons. **(A)** Schematics summarizing the results obtained by Kuba et al., 2005, 2006, 2014; Kuba, 2012 on bird auditory neurons (see main text for references). Auditory neurons responding preferentially to high-frequency sounds (top) display a compact SD compartment together with a short and distant AIS while low-frequency neurons (bottom) display a larger soma, longer dendrites and a longer AIS located close to the soma. Middle-frequency neurons display an intermediate morphology, both at the SD and AIS levels. **(B)** Schematics summarizing the results obtained by Gulledge and Bravo (2016) using computational modeling. While neurons with a small SD compartment display a higher excitability when the AIS is of intermediate length and/or close to the soma, larger neurons are more excitable when the AIS is longer and/or further away from the soma.

We already mentioned that, in the bird auditory nucleus laminaris, AIS geometry was correlated with the neuron preferred frequency in a manner consistent with the theoretical predictions (Kuba et al., 2006). Since the nucleus laminaris is tonotopically organized, such that neuronal position correlates with the preferred frequency encoded (with high-frequency and low-frequency neurons located in the rostromedial and caudolateral regions, respectively; Rubel and Parks, 1975), it means that AIS geometry depends on the neuron position within the nucleus (Kuba, 2012; Kuba et al., 2014). Interestingly, the tonotopic organization of the nucleus is also associated with a gradient in dendritic morphology (Smith and Rubel, 1979; Kuba et al., 2005; Kuba, 2012). Specifically, dendritic arborization length seems to be negatively correlated with preferred frequency such that high-, middle- and low-frequency neurons exhibit a small, medium and large dendritic arbor, respectively (Smith and Rubel, 1979; Kuba et al., 2005; Kuba, 2012). While the influence of soma size has been less studied, some observations suggest that the soma surface is also negatively correlated with preferred frequency (Kuba et al., 2005). This SD scaling seems to favor the integration of fast inputs and improve interaural time-detection sensitivity in high- and middle-frequency neurons as they are more electrotonically compact than low-frequency neurons (Kuba et al., 2005). Consistently, this tonotopic gradient of dendritic complexity across the nucleus laminaris is also associated with a gradient of EPSC filtering: more filtering occurs in low-frequency neurons due to a more complex and less compact dendritic tree leading to smaller and slower somatic EPSCs. On the other hand, EPSCs are larger and faster in the compact middle and high-frequency neurons (Kuba et al., 2005; Slee et al., 2010). In this scheme, the optimal output response of the neuron seems to depend mainly on the passive properties of the SD compartment. Indeed, other studies have suggested that higher interaural time-detection sensitivity and noise tolerance was achieved with a passive somatic compartment (Ashida et al., 2007) and that restricting the invasion of the AP in the dendrites could be necessary to avoid distortion in synaptic integration of high-frequency inputs (Scott et al., 2007). Thus, in auditory neurons, AIS geometry and SD morphology seem to co-vary (Figure 6A), such that the optimal detection of specific frequencies is achieved through the synergistic influence of SD compactness and AIS length and distance from the soma.

Another case of axonal and SD co-tuning has been recently reported in neocortical L5 pyramidal neurons, where the axon can arise directly from the soma or from a basal dendrite (Hamada et al., 2016). In this cell type, the distance between the AIS and the soma can vary from 1 to 20 μm and is negatively correlated with the diameter of the apical dendrite. While this may seem unfavorable for spike initiation, this co-scaling seems to stabilize the amplitude of the back-propagating AP recorded at the soma and may represent a homeostatic mechanism stabilizing neuronal output in the face of cell-to-cell variations in morphology (Hamada et al., 2016).

In an elegant computational study, Gulledge and Bravo (2016) specifically investigated the respective influence of the morphological and biophysical properties of the AIS and the SD compartment on neuronal excitability, their results suggesting that optimal neuronal output can only be achieved by coordinated regulation of both compartments. Using simplified and real-morphology models of several neuronal types (Purkinje neuron, dentate granule cells, neocortical and hippocampal pyramidal neurons), the authors demonstrated that the optimal length and location of the AIS is strongly dependent on SD size. In fact, smaller neurons tend to be more excitable when the AIS is medium in length and relatively close to the soma while in bigger neurons excitability is enhanced by a longer and more distal AIS (Figure 6B, Gulledge and Bravo, 2016). In addition to this general insight, this computational study also suggests that modifications in AIS length (and the associated sodium channel content) should have more impact than AIS-soma distance on excitability, while changes in SD morphology (and the associated capacitive behavior) are more critical than changes in SD ion channel density (Gulledge and Bravo, 2016). Interestingly, using models of 28 fully reconstructed L5 pyramidal neurons, Hay et al. (2013) reached similar conclusions. To ensure stereotypical activity (matching the experimentally recorded range of firing), their model predicts that the density of ion channels in the axon and soma have to scale linearly with the conductance load of dendritic and somatic surface area (Hay et al., 2013). Although formulated in a different way, the modeling work performed by Brette and colleagues also came to the conclusion that the “current sink” effect of the SD compartment needs to be counterbalanced by AIS location or ion channel content (Platkiewicz and Brette, 2010; Brette, 2013; Telenczuk et al., 2017). As mentioned by Gulledge and Bravo (2016), these results suggest that future studies should look for potential co-variations of AIS architecture, SD morphology and neuronal output. To our knowledge, very few studies have been performed in that direction (Hamada et al., 2016; Moubarak et al., 2019).

It is noteworthy that all the studies cited in this section have been performed on neuronal types exhibiting what we previously named a “classical polarity,” i.e., with fairly passive dendrites. Depending on their morphology, passive dendrites can constitute a major impediment to neuronal excitability that may be overcome by modifications of AIS geometry or ion channel content. Unsurprisingly, we will see that other neuronal types with a higher SD excitability may not follow the same rules.

### Absence of Co-tuning: When Excitable Dendrites Make Neurons Robust to AIS Variations

**Figure 7:**
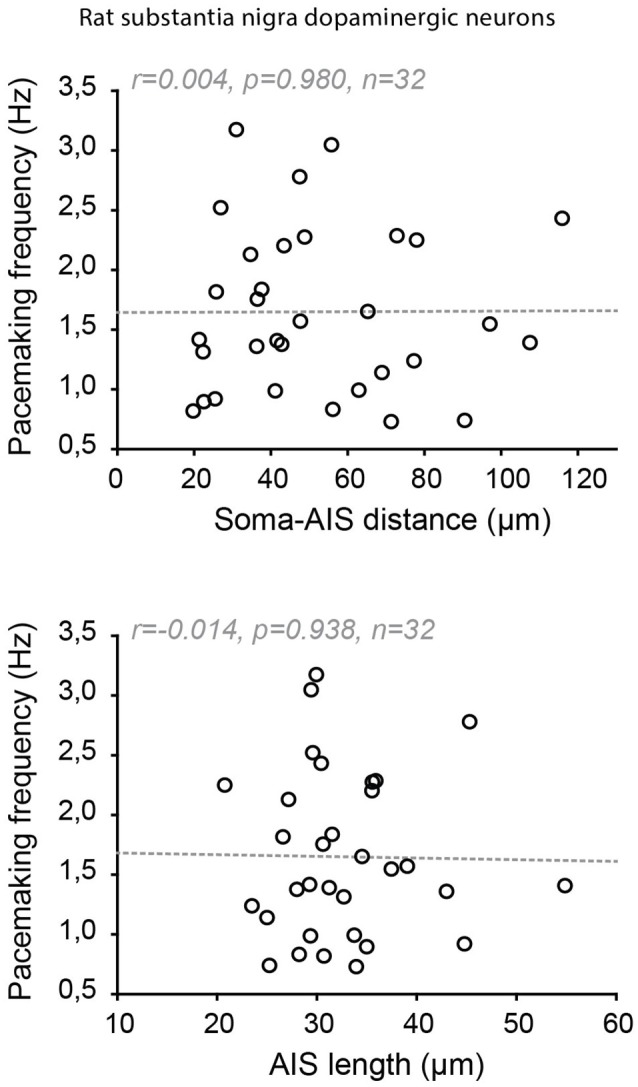
Absence of co-tuning of AIS geometry and firing frequency in rat dopaminergic neurons of the substantia nigra pars compacta. Scatter plots showing the absence of relationship between soma-AIS distance (top) or AIS length (bottom) and pacemaking frequency in 32 rat neurons recorded *in vitro*. Reproduced from Moubarak et al. (2019).

Indeed, significant cell-to-cell variations in AIS geometry have been reported in neuronal types that faithfully back-propagate APs (Hausser et al., 1995; Martina et al., 2000; González-Cabrera et al., 2017; Meza et al., 2018; Moubarak et al., 2019). In the oriens-alveus interneurons, the soma-AIS distance was found to vary between 0 and ~120 μm, the axon arising either from a subiculum- or CA3-oriented dendrite (Martina et al., 2000). This is a considerable range when we consider that variations under 20 μm in pyramidal neurons are correlated with (and compensated by) changes in apical dendrite diameter (Hamada et al., 2016). In spite of this range of variation, no obvious difference in output or in AP back-propagation was noticed between the neurons with a close or a remote AIS (Martina et al., 2000). In the substantia nigra dopaminergic neurons, it was noted early on that the axon could be located at distances from the soma exceeding 200 μm in the adult rat (Hausser et al., 1995). More recent studies performed on juvenile rats and mice have specifically measured the cell-to-cell variations in AIS geometry and their potential influence on neuronal output (González-Cabrera et al., 2017; Meza et al., 2018; Moubarak et al., 2019). Similar to what was observed in oriens-alveus interneurons, the AIS-soma distance was found to vary between 20 and 125 μm in the rat (Moubarak et al., 2019) and 10–70 μm (Meza et al., 2018) or 15–100 μm in the mouse (Goaillard, Moubarak, Tapia and Tell, unpublished observations). Interestingly, in rat dopaminergic neurons, neither variations in AIS distance nor in AIS length seemed to be associated with changes in neuronal output (AP back-propagation, AP shape, firing frequency; Figure 7; Moubarak et al., 2019). Measurements of sodium currents in the SD compartment and real-morphology modeling based on 37 fully reconstructed neurons suggested that SD excitability plays a predominant role in neuronal output, such that variations in AIS geometry are tolerated. On the other hand, this study also predicted that cell-to-cell variations in SD morphology may strongly influence firing frequency, although no simple relationship between these two parameters could be detected (Moubarak et al., 2019). The results concerning a potential relationship between AIS geometry and firing frequency are more contrasted in mouse dopaminergic neurons: while *in vitro* measurements of AIS geometry and firing frequency confirmed the lack of relationship between these variables (Goaillard, Moubarak, Tapia and Tell, unpublished observations), results obtained *in vivo* showed that AIS length was correlated with firing frequency (Meza et al., 2018). The presence of synaptic activity *in vivo* and the sensitivity of both ligand-gated and voltage-gated ion channels to anesthetics may explain the differences observed between these studies.

## Open Questions

In this review, we presented examples demonstrating that mammalian neurons use a wide variety of dendro-axonal solutions to generate APs and release neurotransmitters onto their post-synaptic targets. However, from these observations, it is difficult to extract a general rule that would relate dendritic and axonal morphologies and excitabilities. At one end, auditory neurons in birds seem to rely on an “optimal” co-tuning of SD morphology and AIS geometry that allow them to encode specific sound frequencies and interaural time differences (Figure 6A). In pyramidal neurons, the co-scaling of apical dendrite diameter and AIS distance from the soma seems to stabilize neuronal output (Hamada et al., 2016). However, in most other cases, it is difficult to see the variations in dendritic and axonal morphologies as “optimal” solutions for neuronal activity. For instance, the SD morphology of dopaminergic neurons is extremely variable and independent of AIS geometry and is not really predictive of neuronal output (Moubarak et al., 2019). This lack of link between neuronal output and morphology could be due to several reasons. On one hand, neuronal morphology might not be sufficient to predict output because many other variables, including the heterogeneous densities of a variety of ion channels located in the soma, dendrites and AIS, need to be included in the equation (Weaver and Wearne, 2008; Moubarak et al., 2019; Otopalik et al., 2019). Another possibility is that neuronal morphology does not need to be “optimal” but just good enough for a given neuronal type. Indeed, in two recent studies, Otopalik et al. (2017a,b) demonstrated that crustacean motoneurons seem to be rather insensitive to variations in their dendritic morphology, such that synaptic integration is relatively stable in the face of cell-to-cell variations in morphology. This led the authors to postulate that the relationship between morphology and neuronal output might be many-to-one (Otopalik et al., 2017a,b), meaning that the same output can be produced by neurons with vastly different morphologies. As the specific biophysical properties of dendrites were not assessed in these studies, this many-to-one relationship could also hide some co-variations of dendritic morphology and biophysical properties, such that ion channel expression partly compensates for morphological variations. In fact, other studies performed on the development of crustacean motorneurons have elegantly demonstrated that neuronal output remains stable despite massive SD growth, suggesting that biophysical properties must change along with morphological growth to maintain neuronal output (Bucher et al., 2005). Even though computational simulations have suggested that neuronal activity might be more sensitive to morphological parameters than to biophysical ones (Weaver and Wearne, 2008; Moubarak et al., 2019), an interesting hypothesis is that the specific influence of dendrites and axon on neuronal output can only be understood if the biophysical properties and morphologies of each compartment are measured in each neuron together with its specific pattern of activity. In summary, the current state of knowledge suggests that, even within a same neuronal type, there might be many dendro-axonal morphological solutions allowing to produce the same output (Samsonovich and Ascoli, 2006), just as many biophysical solutions have been demonstrated to produce the same output (Prinz et al., 2004; Marder and Goaillard, 2006; Schulz et al., 2006; Taylor et al., 2009).

Across neuronal types, we showed that the division of labor between axons and dendrites can also take multiple forms associated with strong morphological variations, some neuronal types carrying out all functions (AP initiation and propagation, neurotransmitter release) in the absence of a clearly defined axon. Even though it is difficult to easily explain these observations, other constraints than “optimal” information processing might need to be considered. For instance, many studies have been dedicated to understanding how the wiring of neuronal networks are optimized (Cherniak, 1994; Rivera-Alba et al., 2014), and how optimal circuit architecture might impose specific constraints on neuronal morphology. Rivera-Alba et al. (2014) suggested that cell body externalization (sometimes leading to unipolar neuronal morphologies) might occur because it drastically reduces the length of cable (dendrites and axons) that need to be produced. The energetic cost of circuit function might also need to be considered (Attwell and Laughlin, 2001; Niven, 2016). For instance, dissociated dendritic (local) and axonal (distal) neurotransmitter release might represent an economical alternative to interneuronal populations for short-range lateral inhibition.

## Conclusion

In conclusion, many neuronal types in the mammalian nervous system do not comply with the classical polarity scheme that drove much of our understanding of information processing at the single-neuron level. Evidence suggests that classical “axonal” functions can be carried out by dendrites with peculiar biophysical properties and that the rule balancing dendritic and axonal morphologies and excitabilities might vary from one neuronal type to another. Even though it is unlikely that the properties of these compartments are independently regulated within one given neuronal type, the diversity of schemes and the wealth of parameters involved currently prevent us from easily understanding the logic that relates variations in axonal morphology/excitability to variations in dendritic morphology/excitability. Nonetheless, it is clear that mammalian neurons are using a tremendous diversity of solutions to carry out a similar function, initiating and propagating APs, and releasing neurotransmitters.

## Author Contributions

J-MG, EM, MT and FT wrote the manuscript.

## Conflict of Interest

The authors declare that the research was conducted in the absence of any commercial or financial relationships that could be construed as a potential conflict of interest.
